# Prevalence of Surgical Site Infections Following Coronectomy: A Systematic Review and Meta-Analysis

**DOI:** 10.3390/dj12120379

**Published:** 2024-11-23

**Authors:** Evangelos Kostares, Georgia Kostare, Michael Kostares, Athanasios Tsakris, Maria Kantzanou

**Affiliations:** 1 Department of Microbiology, Medical School, National and Kapodistrian University of Athens, 115 27 Athens, Greece; gioulikostare@gmail.com (G.K.);; 2 Department of Anatomy, Medical School, National and Kapodistrian University of Athens, 115 27 Athens, Greece; michkostares@gmail.com (M.K.); atsakris@med.uoa.gr (A.T.)

**Keywords:** surgical site infections, SSI, mandibular third molar, coronectomy, prevalence, meta-analysis

## Abstract

**Background/Objectives:** This systematic review and meta-analysis aimed to investigate the prevalence of surgical site infections (SSIs) following coronectomy of mandibular third molars. **Methods**: A comprehensive literature search was conducted in Medline, Scopus, Web of Science, and Google Scholar databases up to 30 July 2024. Two independent reviewers performed study selection, data extraction, and quality assessment using the Newcastle–Ottawa Scale. Observational studies assessing SSI prevalence following coronectomy were included. The pooled prevalence of SSI with 95% confidence intervals (CI) was calculated using a random-effects model. Heterogeneity was assessed using the I^2^ statistic, and meta-regression was conducted to explore the influence of continuous variables. **Results**: A total of 22 studies involving 2173 coronectomy procedures were included. The overall pooled prevalence of SSI was 2.4% (95% CI: 1–4.3%), with substantial heterogeneity (I^2^ = 81%). Meta-regression showed no significant effect of the examined variables on SSI prevalence. No study was identified as a significant outlier. Quality assessments revealed that all studies had moderate methodological quality. **Conclusions**: Considerable heterogeneity was observed, likely due to variations in study settings, geographical regions, and timeframes, among other factors. Therefore, this study underscores the need for further rigorous research to better understand SSI risk factors and enhance management strategies for this postoperative complication.

## 1. Introduction

The mandibular third molar typically develops between the ages of 8 and 15 years and erupts approximately between the ages of 18 and 24 years. However, third molars are the most frequently impacted teeth, with 17% to 69% of cases presenting some degree of impaction [1]. Impaction can be either complete or partial, often involving coverage by another tooth, bone, or soft tissue. Several factors contribute to impaction, and numerous theories have been proposed over the decades. Impaction is predominantly associated with abnormal developmental growth of the jaw and teeth, which can lead to a lack of space, an aberrant eruption path, abnormal positioning of the tooth bud, or pathological lesions [2,3]. Regardless of whether one or several factors contribute to impaction, these teeth, particularly mandibular third molars, often require extraction. The primary reason for extraction is pericoronitis, though other reasons include unrestorable caries, caries in the second molar due to horizontal or mesioangular third molar impaction, cysts, tumors, and more [1].

As with any surgical procedure, various intraoperative and postoperative complications may arise. Common postoperative sequelae such as swelling, pain, and trismus are usually transient; however, they should only be classified as complications if they persist beyond the expected recovery period. Intraoperative complications can include excessive bleeding, damage to the second molars, injury to adjacent soft or hard tissues (including bone fractures), mandibular fractures, and root fractures. Postoperative complications, which may not manifest immediately, can include sensory impairment of the inferior alveolar nerve (IAN) or lingual nerve, as well as postoperative dry socket (DS) and surgical site infections (SSIs), with prevalence rates of 6.7% and 1.7%, respectively [4,5,6,7,8].

One of the most severe complications arises when the roots of the third molar are in close proximity to the IAN, potentially leading to sensory issues, such as temporary or permanent loss of sensation or altered sensation in the lower cheek, lip, teeth, and gingivae, which are innervated by this nerve. In cases where IAN damage is a concern, a procedure known as coronectomy may be performed. This involves sectioning and removing the crown of the tooth while leaving the roots in situ. Indications for coronectomy include lower wisdom teeth that are radiographically close to the inferior alveolar canal, signs of narrowing or diversion (loop) of the canal, darkening of the roots in the apical third with canal interruption, interruption of the lingual cortical bone, and vital teeth without caries or periodontal or periapical pathology. Several randomized controlled trials have investigated the risk of IAN damage compared to extraction, demonstrating a lower risk with coronectomy [8]. However, coronectomy is still associated with complications such as DS, SSI, re-operation, lingual nerve injury, and IAN injury [1,8]. An SSI, as defined by the Centers for Disease Control and Prevention (CDC), is an infection that occurs after surgery in the part of the body where the surgery took place. SSIs are categorized into three types: superficial incisional (affecting the skin and subcutaneous tissue), deep incisional (involving deeper tissues like fascia and muscle), and organ/space infections (affecting any part of the anatomy that was manipulated during surgery). Symptoms include redness, pain, fever, and drainage from the wound. SSIs can develop within 30 days post-surgery, or up to 90 days if implants are involved [9].

Recent studies have highlighted the ongoing interest in refining surgical techniques to mitigate complications, particularly those related to nerve injuries. For instance, the systematic review by Almohammadi, T. et al. [10] explored microsurgical interventions for managing iatrogenic trigeminal nerve injuries, demonstrating that techniques like direct neurorrhaphy and nerve grafting can significantly improve sensory recovery in patients who suffered nerve damage during oral surgeries. Additionally, Abu-Mostafa, N. et al.’s [11] review emphasized the effectiveness of coronectomy as a safer alternative to the full extraction of impacted mandibular third molars in cases where the risk of inferior alveolar nerve injury is high. Their findings suggest that coronectomy not only reduces the incidence of permanent nerve damage but also has comparable postoperative infection rates to complete extractions. These studies underscore the need for evidence-based surgical techniques to mitigate nerve damage while ensuring patient safety. However, the variability in reported infection rates following coronectomy highlights a critical gap in understanding the prevalence and determinants of SSIs, necessitating further investigation to optimize postoperative outcomes in this context.

The scientific literature shows considerable variation in reported SSI rates following third molar coronectomy. Therefore, the main goal of this ongoing study is to deliver a more accurate and dependable assessment of SSI prevalence after these procedures, along with an investigation of potential risk factors. This refined analysis, achieved through rigorous meta-analytical methods, allows for the systematic consolidation of data from a comprehensive literature review. By providing a robust pooled estimate, this review fills a critical gap in the literature, serving as a valuable reference for clinicians and researchers aiming to optimize postoperative management and patient outcomes. This effort aims to significantly improve our understanding of this particular clinical scenario.

## 2. Materials and Methods

### 2.1. Search Strategy

In accordance with the Cochrane Handbook for Systematic Reviews of Interventions, a thorough literature search was conducted. The PRISMA guidelines were used to structure and report this systematic review. The PRISMA checklist is available in the Supplementary Materials as Supplementary Table S1. A comprehensive literature search was independently conducted by two researchers across Medline (via PubMed), Scopus, Web of Science, and selectively in Google Scholar (for gray literature). The search was conducted up until 30 July 2024, with no time restrictions. The reviewers employed a combination of keywords, including “third molars”, “coronectomy”, and “infect*”. The complete search algorithms for each database can be found in the Supplementary Materials as Supplementary Table S2.

In addition to database searches, the reviewers thoroughly examined the reference lists of all relevant studies to ensure the inclusion of any potentially overlooked articles. All identified studies were meticulously cataloged using the Zotero reference management program (version 6.0.18), where duplicate citations were systematically identified and removed.

The study selection process involved a two-step approach. Initially, the team assessed the titles and abstracts of the identified papers, adhering strictly to predefined inclusion and exclusion criteria, which were based on the PEO framework. A comprehensive list of these criteria is presented in Table 1. In the context of the PEO framework:

Population (P): Adult patients with third molars who underwent coronectomy.

Exposure (E): The exposure of interest was SSI following coronectomy of third molars.

Outcomes (O): The primary outcome was the prevalence of SSIs. Additionally, the review aimed to identify risk factors contributing to SSI.

This review aimed to determine the overall prevalence of SSIs after coronectomy procedures and to identify potential risk factors associated with these infections.

In the subsequent phase, the full manuscripts of the remaining papers were obtained and thoroughly assessed. Any concerns regarding potential omissions in study selection were resolved by consensus among the team members. Finally, for each included study, the following information was gathered: the primary author’s name, publication year, study design, continent of origin, country, study duration, total number of third molars that underwent coronectomy, gender distribution, mean age, and the number of patients who experienced SSI.

### 2.2. Quality Assessment

Two independent researchers thoroughly assessed each study using the Quality Assessment Tools created through a partnership between the Universities of Newcastle, Australia, and Ottawa, Canada. They used the modified Newcastle–Ottawa Scale (NOS) for cross-sectional studies and the original NOS for cohort studies. Finding any possible methodological or survey-related problems that would compromise the research’s internal validity was the goal of this study. Three main criteria were used to score the studies: the choice of study groups, the comparability of those groups, and the identification of exposure or outcome for case–control or cohort studies (or cross-sectional studies using the modified technique). This evaluation was conducted using a “star system”. Studies scoring between 7 and 9 were deemed low risk of bias (high quality), those with scores of 4 to 6 were rated as moderate quality, and scores from 0 to 3 indicated high risk of bias (low quality) [12].

### 2.3. Statistical Analysis

Utilizing RStudio software (version: 2022.12.0 + 353), we conducted statistical analysis, and the meta-analysis, employing the metafor package, estimated pooled prevalence along with 95% confidence intervals (CI) using the DerSimonian and Laird random-effects model. The Freeman–Tukey double arcsine transformation was applied, and visual inspection of the forest plot, along with Cochran’s Q statistic and its *p* value, assessed heterogeneity among studies. The Higgins I^2^ statistic, indicating true heterogeneity magnitude, was calculated with its 95% CI, categorizing values into 0–40%, 30–60%, 50–90%, and 75–100% for not important, moderate, substantial, and considerable heterogeneity, respectively. Identifying influential outlying effect sizes involved screening for externally studentized residuals and leave-one-out diagnostics. Due to considerable heterogeneity, a meta-regression analysis was performed, assessing the year of publication, the proportion of males, and the mean age as moderators. Variables like smoking status, surgery duration, comorbidities, alcohol consumption, obesity, and surgeon expertise were excluded due to insufficient data (fewer than ten studies). Unless specified otherwise, statistical significance was set at *p*  =  0.05 (two-tailed). To assess publication bias qualitatively in the context of comparative data, methods such as Egger’s test, Begg’s test, and funnel plots are often used. However, in this meta-analysis of proportions, there is a lack of clarity or consensus on defining positive results [13]. Consequently, these tests were not employed, and a qualitative approach was instead adopted for evaluating publication bias.

## 3. Results

### 3.1. Results and Characteristics of the Included Studies

**Figure 1:**
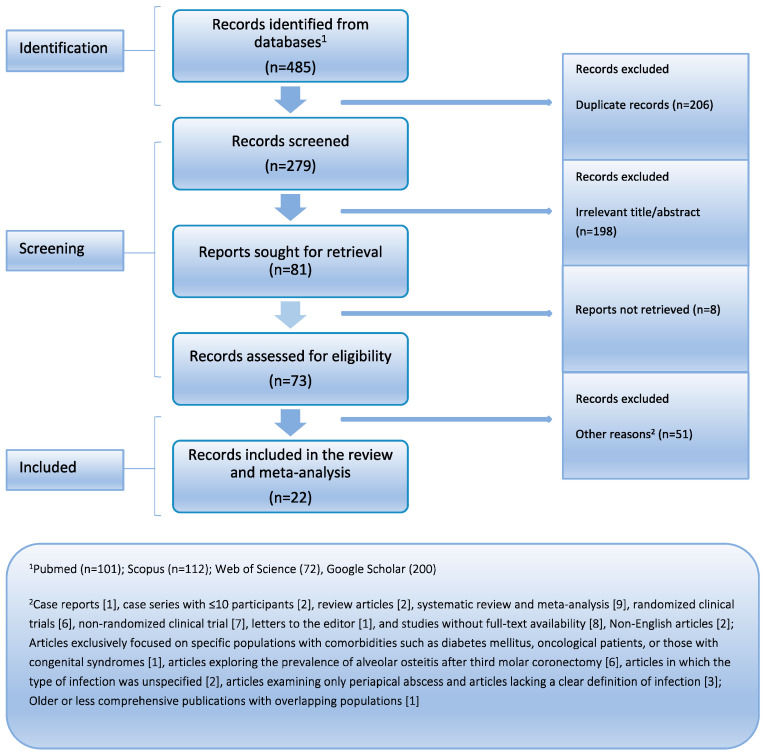
Visual representation illustrating the methodical process of identifying and selecting pertinent studies in the search results.

Initially, 485 records were identified across multiple databases. After removing duplicates, 198 records were excluded based on irrelevant titles and abstracts. Full-text assessment led to further exclusions, resulting in the final selection of 22 eligible studies for analysis. Figure 1 presents the PRISMA flowchart. Every article was released in the years 2004 to 2024. Among these, eight studies were cohort designs, ten were cross-sectional designs, three were case series, and one was a case–control study. Geographically, the majority of these investigations were conducted in various parts of Asia (*n* = 12, including Japan, India, China, Israel, Hong Kong, Turkey, South Korea, and Nepal), followed by Europe (*n* = 9, including Spain, the UK, Italy, the Netherlands, and Denmark) and South America (*n* = 1, Brazil). On average, males constituted 34.4% of the study participants, while the ages of participants ranged from 28 to 33.8 years, with a median age of 27.1 years. Finally, it is noteworthy that all these studies were assessed as having moderate quality, indicating a moderate risk of bias in their findings. The descriptive attributes of the studies included in this analysis are meticulously presented in Table 2.

### 3.2. Prevalence of SSIs Following Coronectomy of Third Molars

**Figure 2:**
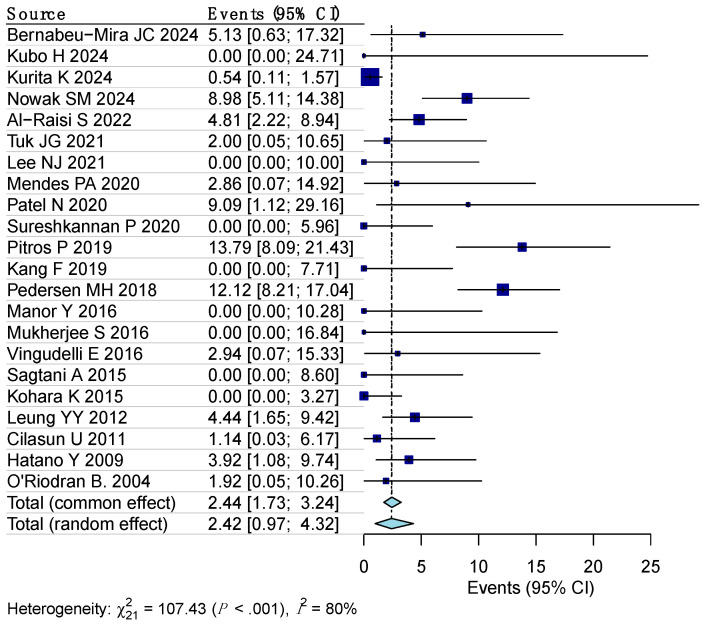
Forest plot examining the determined prevalence of SSIs following coronectomy of third molas utilizing a random-effects model [7,14,15,16,17,18,19,20,21,22,23,24,25,26,27,28,29,30,31,32,33].

The pooled prevalence of surgical site infections (SSIs) following third molar coronectomy was estimated using a random-effects model, revealing a prevalence rate of 2.4% (95% CI: 1–4.3%). As illustrated in Figure 2, substantial heterogeneity was observed among the included studies, with an I^2^ value of 81% (95% CI: 54–87%, *p* < 0.001). To assess the impact of individual studies on the overall results, influence diagnostics were conducted. Detailed findings, along with a forest plot from a leave-one-out sensitivity analysis, are available in the Supplementary Materials (Supplementary Figure S1 and Figure S2). This analysis confirmed that no single study had an undue influence on the pooled estimate.

### 3.3. Meta-Regression Analysis

The meta-regression analysis, as detailed in the Supplementary Materials (Supplementary Table S3), reveals that continuous variables such as the year of publication, the proportion of male participants, and the mean age of patients do not significantly impact the overall prevalence of SSIs following coronectomy of mandibular third molars.

## 4. Discussion

This systematic review provides valuable insights into the prevalence of SSIs following the coronectomy of mandibular third molars. However, the limited availability of comparative data in the existing literature makes it challenging to directly evaluate our pooled estimate against previous findings. The prevalence reported in the available observational studies varies widely, reflecting the diverse methodologies and populations studied. Our analysis demonstrates a 2.4% (95% CI 1–4.3%) prevalence of SSI following the coronectomy of third molars, though this finding is based on studies with considerable heterogeneity. Notably, the meta-regression analysis indicates that factors such as the year of publication, the proportion of male participants, and the mean age do not significantly influence the prevalence of SSI. These results suggest that the SSI rate of 2.4% observed in our study is robust and stable across different demographic and temporal factors, providing a reliable benchmark for future research in this area.

The prevalence of SSIs following coronectomy is likely influenced by a variety of significant factors. These factors include the subjective nature of SSI diagnosis by healthcare providers, the specific surgical techniques employed, and the necessity for additional surgical interventions, which may prolong the duration of the operation. Furthermore, the lack of standardized guidelines for antibiotic administration, along with patient-related risk factors such as diabetes, obesity, age, gender, oral hygiene practices, and habits like tobacco and alcohol use, are crucial contributors. Additionally, the variability in prevalence estimates is generally expected due to differences in the timeframes and geographic locations where the studies were conducted. Therefore, it is crucial to remember that in a proportionate meta-analysis, a high I^2^ score does not always indicate inconsistent data [13].

Regarding alternative therapies, Cervera-Espert et al. [35] conducted a comprehensive meta-analysis using data from four separate studies and concluded that there is not enough statistical support to declare with confidence that coronectomy lowers the incidence of infections (Odds Ratio = 0.87, 95% CI 0.41–1.84) (I^2^ = 0%, *p* = 0.539). Pitros P. et al. [36] conducted a comprehensive evaluation of four studies and found that the infection rates for surgical extraction and coronectomy varied. Infection rates following surgical extraction varied from 0% to 6.7%, but those following coronectomy ranged from 1% to 5.8%. According to these data, compared to surgical extraction, coronectomy does not appear to provide a statistically significant decrease in infection rates. According to the findings of another systematic analysis by Hounsome et al. [37], preventative removal of impacted mandibular third molars may be more cost-effective than retention and routine treatment, even if there is little data to support this claim. Furthermore, due to a lack of data, Ghaeminia H. et al. [38] were unable to reach a firm conclusion in their study on the surgical removal or retention of asymptomatic impacted third molars. They pointed out that frequent clinical evaluations had to be carried out at predetermined intervals if retention is decided upon as the course of action.

This study had some limitations. Considerable unidentified heterogeneity persisted, which suggests that the findings should be approached with caution. The varied outcomes were expected given the nature of these studies [39,40]. Diagnosing SSIs involves subjectivity, and various risk factors—such as diabetes, prolonged surgery, oncology cases, obesity, patient demographics, additional surgeries, lifestyle habits like smoking and alcohol consumption, oral hygiene, and inconsistent antibiotic protocols—may influence the reported SSI rates after third molar coronectomy. The study did not follow the CDC’s cut-off for SSI due to difficulties in arranging extended follow-up, especially in private practice environments. A lack of sufficient studies on this topic also led to the inclusion of cases with follow-ups shorter than one month. Lastly, our meta-analysis was not registered in PROSPERO, which may be a source of reporting bias. Therefore, these results should be interpreted with caution, and further robust studies are necessary. Additionally, the available observational studies were only from Europe, Asia, and South America, all written in English, which may introduce geographical and reporting biases.

This study provides a crucial baseline for understanding the prevalence of SSI following coronectomy, with an observed rate of 2.4%. By consolidating current evidence, it serves as a foundational resource for clinicians and researchers, enabling a more informed approach to managing the risk of SSIs in coronectomy procedures and laying the groundwork for future research. Notably, it underscores the need for high-quality randomized controlled trials (RCTs) to explore the effectiveness of antibiotic prophylaxis and patient management strategies. Such studies will help establish evidence-based guidelines, improve patient outcomes, and standardize practices in the field.

## 5. Conclusions

In conclusion, our study provides a comprehensive meta-analysis of SSIs following coronectomy of mandibular third molars, revealing an overall prevalence of 2.4%. Despite the considerable heterogeneity observed, our findings offer a reliable benchmark for future research. However, the study’s limitations, including the variability in SSI diagnosis and the lack of standardized surgical protocols, underscore the necessity for further research to enhance our understanding of risk factors and develop more effective management strategies for this postoperative complication.

## Figures and Tables

**Table 1:** Inclusion and exclusion criteria.

Criteria	Inclusion	Exclusion
Study Types	Observational studies	Case reports, case series with ≤10 participants, review articles, systematic review and meta-analyses, randomized clinical trials, non-randomized clinical trial, animal studies, letters to the editor, books, expert opinions, conference
Language	Εnglish	Non-English articles
Publication Date	No restrictions	None
Study Design	Studies specifically examining the prevalence of SSIs following third molar coronectomy.	Articles exclusively focused on specific populations with comorbidities such as diabetes mellitus, oncological patients, or those with congenital syndromes, articles exploring the prevalence of alveolar osteitis after coronectomy of third molars, articles in which the type of infection was unspecified, preprint articles, articles examining only periapical abscess, and articles lacking a clear definition of infection were excluded
Population Overlap	Most recent or comprehensive publication used if populations overlap	Older or less comprehensive publications with overlapping populations

**Table 2:** Detailed characteristics of the studies included in the analysis.

First Author	Year of Publication	Study Design	Continent of Origin	Country	Study Period	Third Molars	Proportion of Males (%)	Mean Age (Years)	SSI
Bernabeu-Mira JC [14]	2024	Case-series	Europe	Spain	2011–2022	39	44.1	36	2
Kubo H [15]	2024	Cross-sectional	Asia	Japan	NA	13	30.8	30.7	0
Kurita K [16]	2024	Cohort	Asia	Japan	2005–2020	555	31.7	33.6	3
Nowak SM [17]	2024	Cross-sectional	Europe	UK	2017–2022	167	22	32	15
Al-Raisi S [7]	2022	Cross-sectional	Europe	UK	2017–2020	187	26.2	30	9
Tuk JG [18]	2021	Cross-sectional	Europe	The Netherlands	2019	50	26	NA	1
Lee NJ [19]	2021	Cross-sectional	Asia	South Korea	2016–2018	35	30.8	27.1	0
Mendes PA [20]	2020	Case-series	South America	Brazil	2015–2017	35	23.8	24.3	1
Patel N [21]	2020	Case-series	Europe	UK	2012–2017	22	50	NA	2
Sureshkannan P [22]	2020	Cohort	Asia	India	2017–2019	60	NA	NA	0
Pitros P [23]	2019	Cross-sectional	Europe	UK	NA	116	NA	NA	16
Kang F [24]	2019	Cohort	Asia	China	2013–2017	46	NA	NA	0
Pedersen MH [25]	2018	Cross-sectional	Europe	Denmark	2005–2016	231	NA	NA	28
Manor Y [26]	2016	Cohort	Asia	Israel	2009–2014	34	NA	NA	0
Mukherjee S [27]	2016	Cohort	Asia	India	2012–2016	20	72.2	27.6	0
Vingudelli E [28]	2016	Cohort	Europe	Italy	2011–2012	34	30	28	1
Sagtani A [29]	2015	Cross-sectional	Asia	Nepal	2012–2013	41	NA	NA	0
Kohara K [30]	2015	Cross-sectional	Asia	Japan	2005–2009	111	31.5	33.8	0
Leung YY [31]	2012	Cohort	Asia	Hong Kong	2006–2008	135	35.7	NA	6
Cilasun U [32]	2011	Cohort	Asia	Turkey	2006–2008	88	NA	NA	1
Hatano Y [33]	2009	Case-control	Asia	Japan	2006–2007	102	26.5	32.4	4
O’Riodran B [34]	2004	Cross-sectional	Europe	UK	NA	52	NA	NA	1

NA: not applicable.

## Data Availability

Literature and Rstudio data are available from the corresponding author on reasonable request.

## Supplementary Materials

The following supporting information can be downloaded at: https://www.mdpi.com/article/10.3390/dj12120379/s1, Figure S1: Visual representation of the influence diagnostics for each of the included studies regarding the prevalence of SSI after coronectomy of third molars; Figure S2: Forest plot displaying the re-calculated pooled effects, with one study omitted each time, using the leave-one-out method; Table S1: PRISMA checklist; Table S2: Literature search; Table S3: Meta-regression analysis.
